# Racial/ethnic differences in the association of lifestyle factors with biological aging in NHANES, 1999-2018

**DOI:** 10.1093/gerona/glaf194

**Published:** 2025-09-10

**Authors:** Talha Arif, Aline Thomas, Daniel W Belsky, Yian Gu

**Affiliations:** Vagelos College of Physicians and Surgeons, Columbia University, New York, New York, United States; Taub Institute for Research on Alzheimer’s Disease and the Aging Brain, Columbia University, New York, New York, United States; Department of Neurology, Columbia University, New York, New York, United States; Robert N. Butler Columbia Aging Center, Columbia University, New York, New York, United States; Department of Epidemiology, Joseph P. Mailman School of Public Health, Columbia University, New York, New York, United States; Taub Institute for Research on Alzheimer’s Disease and the Aging Brain, Columbia University, New York, New York, United States; Department of Neurology, Columbia University, New York, New York, United States; Department of Epidemiology, Joseph P. Mailman School of Public Health, Columbia University, New York, New York, United States; Gertrude H. Sergievsky Center, Columbia University, New York, New York, United States

**Keywords:** Racial disparities, Biological aging, Mediterranean diet, Physical activity, Lifestyle factors

## Abstract

Racial and ethnic disparities in healthy aging represent an emerging public health crisis that will only grow worse as our population grows older. Healthy lifestyle behaviors are proposed as a key strategy to promote healthy aging. However, the potential of lifestyle interventions to address aging health disparities is uncertain. We analyzed data from 42 625 adult participants (aged 20-85 years) participating in National Health and Nutrition Examination Survey (NHANES), 1999-2018 to evaluate relationships among healthy lifestyle behaviors and biological aging across White, Black, and Hispanic-identifying groups. We measured healthy lifestyle as adherence to a Mediterranean diet and level of leisure-time physical activity using established methods. We measured healthy aging using the PhenoAge biological age algorithm applied to blood chemistry data. We tested associations within each race/ethnic identity group and compared associations across groups using regression models with interaction terms. We found that within each race/ethnic identity group, greater adherence to a Mediterranean diet and higher levels of leisure-time physical activity were associated with younger biological age, independent of demographic and socioeconomic confounders, obesity, and smoking. However, these associations were stronger among White- as compared to non-Hispanic Black- and Hispanic-identifying adults. Results suggest that healthy lifestyle factors are likely to promote healthy aging across the population. However, lifestyle factors alone may not be sufficient to completely address race/ethnic disparities in healthy aging. Future studies will need to investigate additional ways to reduce racial and ethnic disparities in healthy aging.

## Introduction

Biological aging is the progressive loss of integrity and resilience capacity across cells, tissues, and organs that occurs as we grow older, ultimately causing disease, disability, and death.^1^ The Geroscience Hypothesis proposes that interventions to slow or reverse biological processes of aging can delay or prevent aging-related disease, increasing the period of life lived in good health.^2^ Much effort to test the Geroscience Hypothesis focuses on pharmaceutical interventions.^3^^,^^4^ This focus has raised concerns that interventions to modulate biological aging will primarily benefit the wealthiest and most privileged in society. However, biological processes of aging are also affected by environmental factors and individuals’ behaviors.^5^ This connection integrates the emerging field of geroscience with public health efforts to reduce health disparities.

Race and ethnicity are social constructs that categorize groups of people connected by ancestry and geographic and social origins.^6^ Recent changes in guidelines for reporting race/ethnicity acknowledge that they do not have scientific or biological basis, but can be useful as a tool to study health disparities.^7^ For example, Black and Hispanic Americans experience earlier onset of aging-related disease and shorter average lifespans as compared to White Americans. Environmentally accelerated biological aging thus could represent a modifiable pathway to reduce these aging health disparities.^5^^,^^8–10^ In particular, lifestyle behaviors—such as diet and physical activity—may offer easier-to-implement interventions to modulate the pace of biological aging.^11^^,^^12^ Indeed, there are an increasing number of geroscience trials focused on diet and exercise.^13–15^ To explore the potential of such interventions to contribute to public health efforts to address health disparities, we investigated how these health behaviors relate to biological aging within self-identified racial/ethnic groups of Americans.

In the current study, we analyzed healthy-lifestyle and biological aging data within the National Health and Nutrition Examination Survey (NHANES), a nationally representative sample of the US population taken every 2 years since 1999. Following the method established in our prior work,^16^ we measured healthy lifestyle from participant reports of adherence to a Mediterranean diet (MeDi), physical activity from time spent in leisure-time physical activity (LTPA), and biological aging from the PhenoAge blood-chemistry algorithm. We examined associations of healthy behaviors with biological aging within race/ethnic groups and tested the racial/ethnic disparities in such associations.

## Methods

### Study design and participants

We combined 10 biennial cross-sectional NHANES data sets from 1999 to 2018 using an established data set composed in a previous study.^16^ NHANES is a nationally representative survey designed to assess the health and nutritional status of the US population. Each biennial survey recruits around 5,000 participants in 15 counties across the country. Demographics, socioeconomic, and lifestyle information were collected during an in-home interview; and dietary interview, medical and physical measurements, and laboratory tests were recorded at a physical examination conducted by trained medical personnel in a mobile examination center. Details of the study design, recruitment procedure, and data collection are available from the US Centers of Disease Control and Prevention. The protocol of NHANES was approved by the National Center for Health Statistics Research Ethics Review Board, and all participants provided written informed consent.

**Figure 1.**
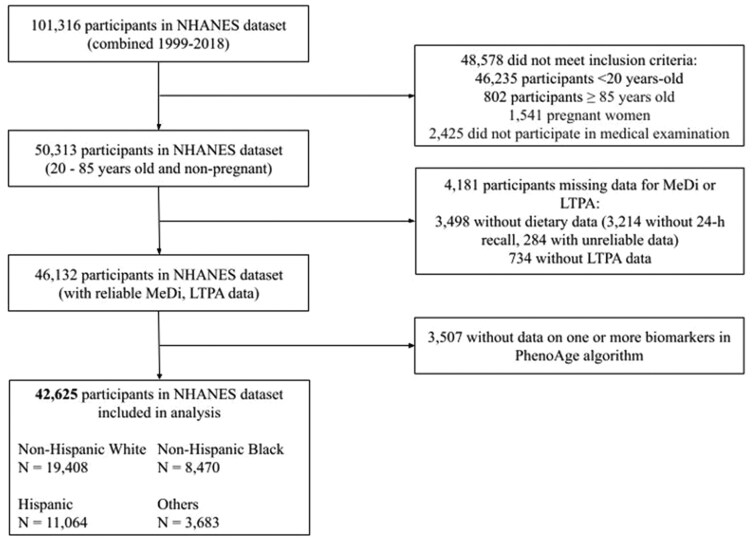
Participant selection flow chart in NHANES 1999-2018.

We included nonpregnant participants aged ≥20 and <85 years old. Participants ≥85 years old were excluded because no exact age was available with ages ≥85 years old recorded as 85 to maximize confidentiality in the survey. Of the 50,313 nonpregnant individuals aged 20-84 years old who were seen at the medical examination, we excluded participants with missing data for diet or LTPA (*n* = 4,181) and blood chemistries (*n* = 3,507). In total, 42,625 participants were included in the analysis (Figure 1).

### Exposure ascertainment

#### Race/ethnicity

Participants self-reported race/ethnicity through the Demographic Section questionnaire,^17^ which includes 4 categories: non-Hispanic White, non-Hispanic Black, Hispanic (including Mexican American), and Others (including Asian and multi-racial respondents).

#### MeDi score

Participants reported dietary information through validated 24-hour dietary recalls delivered by trained dietary interviewers, using the US Department of Agriculture’s MultiPass Method. The MeDi score, reflecting adherence to the traditional MeDi, was calculated from 9 food components in 2 steps. First, we assigned a score of 0 or 1 to each food group using sex-specific medians of energy-adjusted intakes used as cutoff values.^18^ For beneficial components (vegetables, fruits, legumes, cereals, fish, and ratio of monounsaturated to saturated fats), 1 point was given for an intake equal to or greater than the median. For components considered to be detrimental (dairy products and meat), 1 point was awarded for an intake less than the median. For alcohol, 1 point was given for mild-to-moderate consumption (ie, 0-1 drinks per day for female participants and 0-2 drinks per day for male participants). Second, the 9 component scores were summed to compute the final MeDi score. The total MeDi score ranges from 0 to 9, with a higher score indicating greater adherence.

#### Leisure time physical activity level

Self-reported physical activity was collected through 2 different questionnaires depending on the NHANES wave (1999-2006 and 2007-2018), both administered during the medical examination.^16^ The questionnaires recorded the frequencies, durations (in minutes) and intensities of LTPA. The total number of minutes per week for each intensity of LTPA was calculated as the frequency multiplied by the duration. The total moderate-to-vigorous LTPA was then coded in metabolic equivalent of task (MET) minutes per week by multiplying the duration of the activities and the intensity-specific MET scores (4.0 MET for moderate and 8.0 MET for vigorous intensity LTPA, as suggested by the NHANES guidelines). Finally, LTPA was classified into 4 levels according to the 2018 national physical activity guidelines: sedentary (no regular physical activity, ie, 0 MET min/week), low (insufficient regular activity, <500 MET min/week, ie, approximately 2 h/week of moderate LTPA), moderate (500-1000 MET min/week), and high (>1000 MET min/week, ie, approximately 4 h/week of moderate LTPA).^19^

### Biological aging

There is no gold standard to measure biological aging.^20^ The current state-of-the-art is a family of algorithms that integrate data from panels of biochemical markers reflecting the conditions of different organs and systems within the body or their cells.^21^^,^^22^ We measured biological aging using the PhenoAge algorithm, a composite measure integrating information about chronological age and 9 laboratory chemistries and hematological markers (albumin, alkaline phosphatase, creatinine, C-reactive protein, glucose, mean cell volume, red cell distribution width, white blood cell count, and lymphocyte %).^23^^,^^24^ PhenoAge is a well-validated measure outperforming other blood chemistry-based biological age algorithms in predicting mortality, health span, and cardiovascular disease, and has a strong relationship with various measures of multimorbidity.^25^ PhenoAge was implemented in the NHANES data using the “BioAge” R package^24^ with the following modifications: C-reactive protein was omitted because this biomarker was not measured during the 2011-2012 and 2013-2014 waves; HbA1c was substituted for glucose to allow for inclusion of participants who were not fasted at the time of blood draw. We previously validated this alternative version as a comparable predictor of morbidity and mortality to the original PhenoAge.^16^ PhenoAge advancement was computed as the difference between algorithm-predicted biological age and chronological age. Values were standardized to have a mean of 0 and a standard deviation (*SD*) of 1 for analysis. PhenoAge advancement values >0 therefore indicate an advanced state of biological aging and increased risk of diseases and mortality; values <0 indicate delayed biological aging.

### Covariates

Demographic factors obtained from the in-home NHANES interview included self-reported age and sex. Socioeconomic factors included educational level (under high school, high school/some college, and bachelor’s degree or above), marital status (married/cohabitating, divorced/widowed/separated, and never married), and family income-to-poverty ratio (defined as the ratio of family income to the year-specific federal poverty threshold).^26^ Lifestyle factors included smoking status (never [<100 cigarettes in life], former [≥100-lifetime cigarettes but do not smoke now], and current) and body mass index (BMI) category (normal weight [<25 kg/m^2^], overweight [25-30 kg/m^2^], and obesity [≥30 kg/m^2^]). Additional covariates included NHANES wave and total caloric intake from the 24-hour recall (in kcal).

### Statistical analyses

We first compared participants’ characteristics across racial/ethnic groups. Chi-square tests were performed for categorical variables and analysis of variance was conducted for continuous variables.

We tested associations of MeDi and LTPA with PhenoAge advancement using linear regression with model covariates for age, sex, calorie intake, and NHANES year. We first fitted models separately by race/ethnicity. To test differences in associations, we added product terms to regression models evaluating the interaction between race/ethnic identity and lifestyle behavior (Model 1). A significant interaction would indicate the association between MeDi or LTPA and biological age differs by racial/ethnic groups. Stratified analyses by racial/ethnic groups were then performed.

In sensitivity analyses, we added additional covariates to account for socioeconomic differences that might be correlated with lifestyle behaviors (marital status, income-to-poverty ratio, BMI, smoking, and educational level; Model 2); for history of 10 major comorbidities that may affect diet or practice of physical activity (diabetes, hypertension, hypercholesterolemia, stroke, cardiovascular disease, chronic bronchitis, liver condition, pulmonary emphysema, thyroid disease, and arthritis; Model 3); and mutual adjustment for MeDi and LTPA (Model 4). Furthermore, to account for possible reverse causality, we conducted a sensitivity analysis excluding participants with a history of the 10 comorbidities.

To compare effect sizes observed for MeDi and LTPA, we used dichotomized variables in a supplementary analysis. MeDi score was dichotomized into low adherence (ie, MeDi score 0-3) vs moderate/high adherence (ie, MeDi scores 4-5 and 6-9). LTPA was categorized into sedentary (no exercise) versus some exercise (low, moderate, and high levels).

All statistical analyses incorporated NHANES complex survey design via weighting with dietary survey weights (WTDR2D, addressing unequal selection probabilities, nonresponse to survey and dietary component, and including the day of the week of recall) to obtain nationally representative estimates. Analyses were conducted using R Studio (Version 2023.06.0).

## Results

Data from 42,625 participants was nationally representative of 197,323,426 US adults with 45.6% of non-Hispanic White participants, 19.8% of non-Hispanic Black participants, 26.0% of Hispanic participants, and 8.6% from Other race/ethnicity (Table 1).

**Table 1.** Characteristics of the study participants by race/ethnicity, NHANES 1999-2018 (*n* = 42,625).

	Total population	Self-reported race/ethnicity				
		**Non-Hispanic White (*n* = 19** **408)**	Non-Hispanic Black (*n* = 8470)	**Hispanic (*n* = 11** **064)**	Other (*n* = 3683)	*P* value
**Representative population size**	197 323 427	136 912 075	20 450 054	26 810 460	13 150 838	
**Gender (female), No. (%)**	21 357 (51.0)	9576 (50.9)	4303 (53.8)	5663 (49.4)	1815 (50.3)	<.001
**Age, mean (*SD*), years**	47.0 (16.7)	48.9 (16.9)	44.1 (15.9)	41.1 (15.1)	44.1 (15.6)	<.001
**MeDi score, mean (*SD*)**	3.97 (1.6)	3.86 (1.60)	4.00 (1.57)	4.26 (1.57)	4.53 (1.67)	<.001
**MeDi level, No. (%)**						<.001
** Low (scores 0-3)**	15 075 (39.2)	8073 (42.0)	3023 (38.0)	3122 (32.1)	857 (27.8)	
** Moderate (scores 4 and 5)**	18 863 (43.2)	8169 (42.3)	3826 (45.0)	5269 (46.7)	1599 (42.5)	
** High (scores 6-9)**	8687 (17.5)	3166 (15.7)	1621 (17.0)	2673 (21.3)	1227 (29.7)	
**LTPA level, No. (%)**						<.001
** Sedentary**	20 411 (40.8)	8368 (37.8)	4305 (48.6)	6193 (50.2)	1545 (39.8)	
** Low**	6777 (17.4)	3356 (18.3)	1248 (14.9)	1549 (14.4)	624 (18.3)	
** Moderate**	4794 (12.4)	2413 (13.2)	831 (9.9)	1081 (10.0)	469 (12.7)	
** High**	10 643 (29.4)	5271 (30.7)	2086 (26.6)	2241 (25.4)	1045 (29.3)	
**Education, No. (%)**						<.001
** Less than high school**	10 991 (16.3)	2978 (11.0)	2070 (22.8)	5442 (40.6)	501 (12.6)	
** High school or some college**	22 175 (55.6)	11 163 (57.3)	4969 (60.4)	4504 (47.3)	1539 (47.1)	
** Bachelor’s degree or higher**	9417 (28.1)	5255 (31.8)	1419 (16.8)	1103 (12.0)	1640 (40.4)	
**Marital status, No. (%)**						<.001
** Married/cohabiting**	25 783 (63.3)	12 370 (66.3)	3802 (43.1)	7191 (63.0)	2420 (63.7)	
** Divorced/widowed/separated**	9086 (18.4)	4261 (18.4)	2237 (23.7)	2081 (15.9)	507 (14.8)	
** Never married**	7386 (18.4)	2605 (15.3)	2362 (33.2)	1674 (21.1)	745 (21.6)	
**Income-to-poverty ratio, No. (%)**						<.001
** Low**	7725 (13.9)	2498 (9.5)	1795 (25.0)	2881 (28.4)	551 (14.8)	
** Middle**	20 878 (48.9)	9460 (46.8)	4274 (54.6)	5517 (55.9)	1627 (49.3)	
** High**	10 514 (37.2)	6313 (43.7)	1621 (20.4)	1399 (15.7)	1181 (35.9)	
**Smoking status, No. (%)**						<.001
** Never**	22 943 (53.4)	9075 (50.2)	4763 (58.5)	6659 (61.7)	2446 (62.4)	
** Former**	10 621 (25.0)	5881 (28.2)	1583 (15.7)	2519 (19.6)	638 (17.1)	
** Current**	9032 (21.6)	4443 (21.6)	2115 (25.8)	1878 (18.7)	596 (20.6)	
**BMI category, No. (%)**						<.001
** Normal**	12 306 (31.0)	6085 (32.0)	2058 (25.3)	2400 (23.3)	1763 (45.3)	
** Overweight**	14 353 (33.5)	6527 (33.9)	2441 (28.0)	4261 (37.6)	1124 (30.3)	
** Obese**	15 497 (35.4)	6564 (34.1)	3868 (46.7)	4303 (39.1)	762 (24.5)	
**Dietary calories, mean (*SD*), kcal**	2133.5 (894.0)	2153.5 (881.8)	2087.5 (980.1)	2123.7 (913.0)	2016.4 (827.3)	<.001
**NHANES wave, No. (%)**						.13
** 1999**	3410 (8.2)	1547 (8.4)	618 (7.9)	1154 (8.8)	91 (4.9)	
** 2001**	3858 (9.3)	2041 (10.0)	695 (8.7)	1003 (8.5)	119 (5.4)	
** 2003**	3647 (9.2)	1954 (9.8)	698 (9.2)	847 (7.4)	148 (7.1)	
** 2005**	3653 (9.3)	1859 (9.9)	815 (9.8)	841 (7.2)	138 (7.1)	
** 2007**	4931 (10.0)	2394 (10.3)	938 (9.8)	1407 (9.8)	192 (7.7)	
** 2009**	5262 (10.2)	2622 (10.4)	862 (10.3)	1509 (10.1)	269 (9.3)	
** 2011**	4313 (10.5)	1712 (10.2)	1076 (10.7)	855 (11.1)	670 (11.7)	
** 2013**	4675 (10.9)	2114 (10.5)	878 (11.1)	1052 (11.9)	631 (13.4)	
** 2015**	4553 (11.0)	1580 (10.3)	912 (10.9)	1417 (12.2)	644 (15.6)	
** 2017**	4323 (11.2)	1585 (10.2)	978 (11.7)	979 (13.0)	781 (17.7)	
**PhenoAge advancement, mean (*SD*), years**	−3.6 (4.6)	−3.8 (4.5)	−2.4 (5.2)	−3.5 (4.5)	−4.4 (4.8)	<.001
**Standardized PhenoAge advancement, mean (*SD*)**	−0.09 (0.94)	−0.12 (0.91)	0.16 (1.10)	−0.07 (0.91)	−0.25 (0.98)	<.001

Abbreviations: BMI, body mass index; LTPA, leisure-time physical activity; MeDi, Mediterranean diet.

Percentages, means, and SDs are of non-missing values and presented as weighted estimates to account for sampling design. Values were missing for 8.2% of the sample for the income-to-poverty ratio, 1.1% for BMI, 0.9% for marital status, and 0.1% for education and smoking status.

### PhenoAge, MeDi, and LTPA by race/ethnic group

The overall mean age was 47.0 (±16.7) years, and participants’ PhenoAge values were 3.62 years younger than their chronological age (mean PhenoAge advancement = −3.62 [±4.6] years; Figures S1 and S2, see online supplementary material for a color version of these figures). Both age and PhenoAge advancement differed across race/ethnicity groups (*p *< .001 for pairwise *t*-tests comparisons, Table 1). Non-Hispanic White participants were on average chronologically older (mean age = 48.9 [±16.9] years) than non-Hispanic Black participants (44.1 [±15.9] years) and Others (44.1 [±15.6] years), whereas Hispanic participants were younger (41.1 [±15.1] years). However, non-Hispanic Black participants were biologically older (mean PhenoAge = −2.4 [±5.2] years) than Hispanic (−3.5 [±4.5] years), non-Hispanic White (−3.8 [±4.5] years), or Other (−4.4 [±4.8] years) participants. Accordingly, non-Hispanic Black participants had a standardized PhenoAge of 0.16 (±1.10), Hispanic participants of −0.07 (±0.91), non-Hispanic White participants of −0.12 (±0.91), and Others of −0.25 (±0.98).

**Figure 2.**
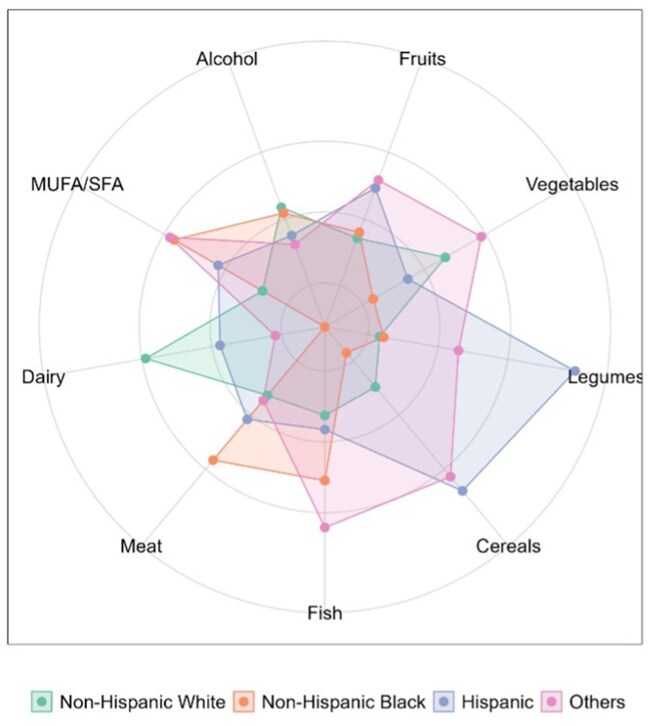
Breakdown of individual MeDi components by race/ethnicity, NHANES 1999-2018 (*n *= 42 625). Spider-chart representation of mean standardized energy-adjusted dietary intakes for the 9 components of the MeDi score. Abbreviations: MeDi, Mediterranean diet; MUFA/SFA, monounsaturated fatty acid/saturated fatty acid ratio.

In our study population, the average MeDi score was 3.97 (±1.6) points out of 9 but differed by race/ethnicity (Table 1). Non-Hispanic White participants had a lower adherence to MeDi with an average score of 3.9 (±1.6), compared to 4.0 (±1.6) for non-Hispanic Black participants, 4.3 (±1.6) for Hispanic participants, and 4.5 (±1.7) for Other participants (*p *< .001). However, looking into the composition of the diet, the contribution of each of the 9 components of MeDi varied across race/ethnicity (Figure 2). Non-Hispanic White participants consumed more vegetables and dairy but less meat and fish compared to non-Hispanic Black and Hispanic participants, whereas Hispanic participants ate more fruits, legumes, and grains, and drank less alcohol than non-Hispanic White and non-Hispanic Black participants. The deviation of PhenoAge from chronological age by tertiles of MeDi is shown in Figure S3 (see online supplementary material for a color version of this figure).

Regarding physical activity, in the total population, 29.4% of the participants had a high level of LTPA, 12.4% a moderate level, 17.4% a low level, and 40.8% were categorized as having a “sedentary” LTPA level. LTPA differed by race/ethnicity: Hispanic participants were more often sedentary (50.2%) than non-Hispanic Black (48.6%), Others (39.8%), and non-Hispanic White participants (37.8%) (*p *< .001). The deviation of PhenoAge from chronological age by tertiles of LTPA is shown in Figure S4 (see online supplementary material for a color version of this figure).

### Association of MeDi with PhenoAge advancement, by race/ethnicity

Greater MeDi adherence is associated with lower PhenoAge across all racial/ethnic groups (Table 2). With every 1-point increase in MeDi score, the PhenoAge advancement was decreased by −0.07 *SD* (95% CI = [−0.08; −0.06], *p *< .001) for non-Hispanic White, −0.04 ([−0.05; −0.02], *p *< .001) for non-Hispanic Black, −0.05 ([−0.06; −0.04], *p *< .001) for Hispanic participants, and −0.12 ([−0.15; −0.09], *p *< .001) for Others.

**Table 2.** Associations of MeDi and LTPA with PhenoAge advancement, estimated by adjusted linear regressions, NHANES 1999-2018 (*n* = 42 625).

	Non-Hispanic White		Non-Hispanic Black		Hispanic		Other	
**A. MeDi and LTPA on PhenoAge in each race/ethnicity group**								
**MeDi score**	β (95% CI)	*P*	β (95% CI)	*P*	β (95% CI)	*P*	β (95% CI)	*P*
** For 1-point**	−0.07 (−0.08; −0.06)	<.001	−0.04 (−0.05; −0.02)	<.001	−0.05 (−0.06; −0.04)	<.001	−0.12 (−0.15; −0.09)	<.001
**LTPA level**								
** Sedentary**	Ref.		Ref.		Ref.		Ref.	
** Low**	−0.25 (−0.29; −0.21)	<.001	−0.11 (−0.18; 0.04)	.002	−0.13 (−0.19; −0.06)	<.001	−0.15 (−0.29; −0.02)	.03
** Moderate**	−0.34 (−0.39; −0.28)	<.001	−0.19 (−0.29; −0.10)	<.001	−0.20 (−0.27; −0.12)	<.001	−0.21 (−0.36; −0.05)	.008
** High**	−0.37 (−0.41; −0.33)	<.001	−0.22 (−0.28; −0.16)	<.001	−0.14 (−0.20; −0.09)	<.001	−0.20 (−0.30; −0.10)	<.001
**B. MeDi-by-race/ethnicity and LTPA-by-race/ethnicity interactions on PhenoAge**								
**MeDi-by-race/ethnicity interaction**	β (95% CI)	*P*	β (95% CI)	*P*	β (95% CI)	*P*	β (95% CI)	*P*
** For 1-point**	Ref.		0.03 (0.01; 0.05)	.001	0.02 (0.003; 0.04)	.02	−0.05 (−0.08; −0.02)	.001
**LTPA-by-race/ethnicity interaction**								
** Sedentary**	Ref.		Ref.		Ref.		Ref.	
** Low**	Ref.		0.14 (0.06; 0.23)	.001	0.13 (0.05; 0.21)	.002	0.10 (−0.04; 0.24)	.15
** Moderate**	Ref.		0.14 (0.04; 0.24)	.005	0.14 (0.05; 0.23)	.002	0.13 (−0.03; 0.29)	.12
** High**	Ref.		0.15 (0.08; 0.22)	<.001	0.23 (0.15; 0.30)	<.001	0.17 (0.06; 0.27)	.002

Abbreviations: LTPA, leisure-time physical activity; MeDi, Mediterranean diet; MET, metabolic equivalent of task; PhenoAge, xxx; Ref., reference.

Panel A presents β coefficients and 95% CIs for the associations between MeDi score or LTPA level and PhenoAge advancement in each of the 4 race/ethnicity groups. The model was adjusted for age, sex, caloric intake, and NHANES year. Panel B presents β coefficients and 95% CIs for the interaction testing the product of MeDi or LTPA by race/ethnicity (with non-Hispanic White as reference) to evaluate whether the associations of MeDi and LTPA with PhenoAge advancement differed across race/ethnicity groups. β coefficients for the interaction of race/ethnicity with MeDi indicate the difference of the MeDi-PhenoAge association between each of the other race/ethnicity groups and the non-Hispanic White (reference) group. For example, the difference of the MeDi-PhenoAge association between non-Hispanic Black and non-Hispanic White participants is −0.04 − (−0.07) = 0.03, suggesting 0.03 years additional younger effect on PhenoAge in non-Hispanic White than in non-Hispanic Black participants for one unit increase in MeDi. β coefficients for the interaction of race/ethnicity with LTPA indicate the difference in the LTPA-PhenoAge association between each of the other race/ethnicity groups and the non-Hispanic White (reference) group. For example, the difference of the low LTPA-PhenoAge association between non-Hispanic Black and non-Hispanic White participants is −0.11 − (−0.25) = 0.14, suggesting 0.14 years additional younger effect on PhenoAge in non-Hispanic White than in non-Hispanic Black participants for the same comparison between low LTPA and being sedentary. PhenoAge advancement was standardized so that regression coefficients are relative to 1 *SD* (= 4.6 years) of PhenoAge advancement. MeDi score was taken as a continuous variable, and LTPA levels were defined as sedentary (0 MET min/week; reference), low (1-500 MET min/week), moderate (500-1000 MET min/week), and high (>1000 MET min/week). Models adjusted for age, sex, total energy intake, and NHANES wave.

The effect sizes of the association between MeDi and PhenoAge were significantly different across race/ethnicity groups (*p* for interaction ≤.02; Table 2, Panel B). The effect size of every 1-point increase in MeDi score on PhenoAge was 0.03 *SD* ([0.01; 0.05], *p *= .001) larger for non-Hispanic White compared to non-Hispanic Black participants, 0.02 ([0.003; 0.04], *p *= .02) larger than Hispanic participants, and 0.05 ([−0.08; −0.02], *p *= .001) smaller compared to Others. Similar results were found using MeDi at 3 categorical levels (data not shown).

### Association of LTPA with PhenoAge advancement, by race/ethnicity

A higher level of LTPA was associated with younger PhenoAge across all racial/ethnic groups (Table 2). Non-Hispanic White participants with a high level of LTPA exhibited a 0.37 *SD* ([−0.41; −0.33], *p *= .001) younger PhenoAge than sedentary non-Hispanic White participants. Similarly, the β coefficients for high LTPA versus sedentary were −0.22 *SD* ([−0.28; −0.14], *p *= .001) for non-Hispanic Black participants, −0.14 ([−0.20; −0.09], *p *= .001) for Hispanic participants, and −0.20 ([−0.30; −0.10], *p *= .001) for Others. For all categories of LTPA (ie, low, moderate, or high levels vs sedentary), non-Hispanic White participants showed significantly greater effect sizes of LTPA on PhenoAge than non-Hispanic Black and Hispanic participants (interaction *p *< .005, Table 2).

### Sensitivity analysis

Further adjustment for socioeconomic factors attenuated the associations of MeDi and LTPA with PhenoAge in all the racial/ethnic groups (Tables S1 and S2). The differential effect by race/ethnicity (ie, interaction term) was also slightly attenuated by adjusting for socioeconomic factors. For example, compared to the low level of LTPA, a high level of LTPA in model 2 was associated with 0.16 *SD* (95% CI = [−0.20; −0.12], *p *< .001) younger PhenoAge in non-Hispanic White and 0.08 ([−0.14; −0.02], *p *< .01) younger PhenoAge in non-Hispanic Black, with a difference of 0.08 *SD* ([0.01; 0.15], interaction *p *< .05) between the 2 races, whereas the difference was 0.15 *SD* ([0.08; 0.22], interaction *p *< .001) between the 2 races in Model 1.

Adjustment for 10 comorbidities and mutual adjustment for MeDi and LTPA did not significantly change the associations. MeDi and LTPA remained significantly associated with PhenoAge for all racial groups.

In analysis excluding participants with these comorbidities (*n* = 10,682 disease-free participants), associations of MeDi and LTPA with PhenoAge were attenuated in varying degrees. Full results are reported in Table S3.

Finally, the associations of MeDi and LTPA with PhenoAge remained significant in both younger and older adults, as demonstrated in sensitivity analyses reported in Figures S3 and S5 (see online supplementary material for a color version of these figures). Further, the correlation of PhenoAge with chronological age by MeDi and LTPA remained significant (Figures S4 and S6, see online supplementary material for a color version of these figures).

### Comparison between MeDi and LTPA effect sizes

**Figure 3.**
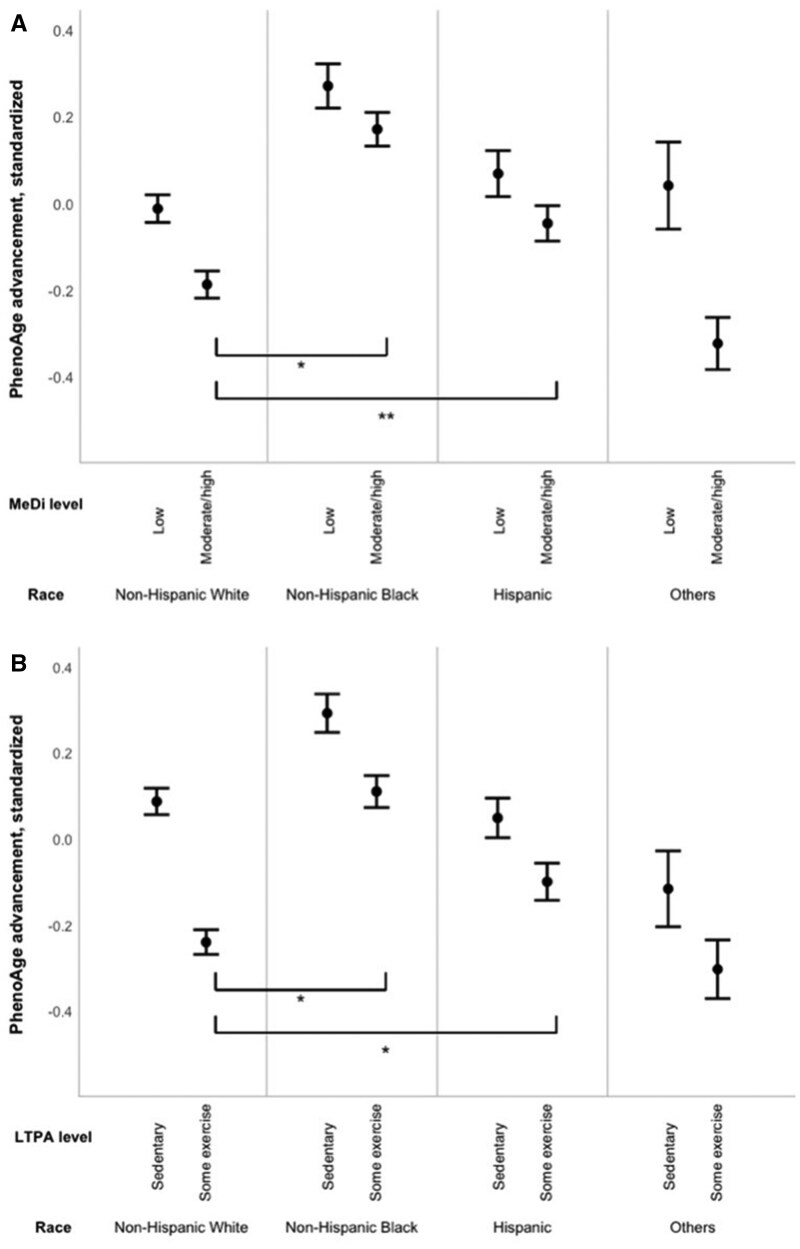
Mean standardized PhenoAge advancement by binary MeDi and race/ethnicity (panel A) and binary LTPA and race/ethnicity (panel B), NHANES 1999-2018 (*n* = 42,625). Marginal means and 95% confidence intervals of the standardized PhenoAge advancement, adjusted for age, sex, total energy intake, and NHANES wave, and weighted to account for sampling design, by categories of MeDi and race/ethnicity (panel A), and LTPA and race/ethnicity (panel B). MeDi level was defined as low (scores 0-3) and moderate/high (scores 4 and 5/6-9), LTPA levels were defined as sedentary (0 MET min/week) and some exercise (≥1 MET min/week). Abbreviations: LTPA, leisure-time physical activity; MeDi, Mediterranean diet; PhenoAge, xxx.*P values were <.05 for the interactions. **P value was .06 for the interaction.

We compared the effect sizes for MeDi and LTPA associations with PhenoAge across race/ethnic groups, using binary variables for exposures (Figure 3). The effect size for some (vs sedentary) LTPA with younger PhenoAge was larger than the effect size for moderate-to-high (vs low) adherence to MeDi, for all race/ethnicity groups, except Others (Table S4). For example, in non-Hispanic White participants, performing some LTPA compared to having no LTPA yielded a −0.33 *SD* (95% CI = [−0.36; −0.29], *p *< .001) younger PhenoAge, whereas a moderate/high adherence to MeDi compared to low adherence yielded a −0.18 *SD* ([−0.21; −0.14], *p *< .001) younger PhenoAge.

## Discussion

We analyzed survey data about diet and exercise and blood-chemistry data on biological age from a series of nationally representative samples of US adults across the period 1999-2018. We found that non-Hispanic Black Americans were biologically older than non-Hispanic White Americans by about one year and a half. This estimate is similar to several previous papers using this measure as well as others.^27–33^ Differences were much smaller between Hispanic and non-Hispanic White Americans.

Furthermore, we found that for all races/ethnicities, healthy behaviors were associated with younger biological age. This finding is a promising indication of the potential of lifestyle factors in yielding beneficial outcomes in aging regardless of race/ethnicity group. However, associations were stronger in non-Hispanic White as compared with non-Hispanic Black and Hispanic participants; the “healthy-aging returns” were about 1.5 times greater for a healthy diet and 2 times greater for physical activity. We consider 3 possible explanations for these findings.

First, if the blood-chemistry algorithm used to measure biological aging was less precise in non-White as compared with White Americans, the excess measurement error would attenuate associations in the non-White group. The biological age measurement we analyzed was developed in the NHANES III sample, which was majority-White. The resulting model could be less precise in non-White Americans. However, the initial validation study of the algorithm did not find evidence of such bias, nor did a follow-up in a separate sample,^9^^,^^33^ although at least one study has reported differences.^32^ Future studies could further explore the extent of the racial composition of samples used to develop measures of biological aging.

Second, if the survey measurements of diet and exercise were less precise in non-White as compared with White Americans, the excess measurement error would attenuate associations in the non-White group. Previous studies have shown that the MeDi may not reflect cultural differences accurately, with limited data on the health benefits of MeDi in minority groups.^34^^,^^35^ In prior studies, the relative contributions of each of the 9 food components to the overall MeDi score vary across racial/ethnic groups.^36^^,^^37^ This was also observed in the NHANES data we analyzed (Figure 2). Diet patterns of non-White NHANES participants may be less well represented by their MeDi scores relative to White participants. Further, the time of day of food consumption may affect impact on biological aging. Studies have demonstrated that time-restricted eating improves circadian rhythm, correlated with reduced morbidity in older adults.^38^ Future studies may need to investigate whether time of food consumption is associated with biological aging and whether this phenomenon differs by race through NHANES. In the case of LTPA, epidemiological evidence, including from NHANES, suggests LTPA is similarly associated with mortality in Black and White Americans,^39^^,^^40^ although some studies found slightly weaker associations in Hispanic participants.^41^ Although few studies have considered racial differences in self-reporting accuracy of LTPA, there is little evidence for systematic differences.^42^ Studies are needed that do not rely on participant self-reports to ascertain diet and activity data to fully exclude measurement error as a cause of the differences we observed.

Third, other risk factors more prevalent among non-White participants may moderate the impact of healthy lifestyle behaviors on biological aging. Previous studies have found various factors such as socioeconomic exposures and psychosocial stress may contribute to the accelerated biological aging in Black as compared with White Americans.^9^^,^^43^^,^^44^ It could be that the effects of these factors on biological aging overwhelm some benefits of healthy lifestyle, resulting in weaker healthy-aging “returns” on healthy behaviors in non-White as compared with White Americans. Additionally, previous studies demonstrate accelerated aging in men compared to women. Although we controlled for sex in all models, and sex difference in biological aging seems to be similar across race/ethnicity groups, at least one study found a marginal increase in aging rate in Black men, arguing for a potential interactional phenomenon between race/ethnicity and sex.^45–47^

Our finding that healthy lifestyle behaviors are significantly associated with lower biological age regardless of race/ethnicity group suggests a universally positive influence of interventions such as MeDi and physical activity in aging. Our finding that this association with biological aging was weaker in non-White as compared with White Americans suggests that, although healthy lifestyle behaviors represent a useful component of efforts to reduce aging disparities, they are insufficient to fully address disparities in healthy aging. One possible path forward is to tailor lifestyle interventions to the specific communities in which intervention is occurring. For example, culturally sensitive interventions to address cardiovascular risk factors in non-Hispanic Black adults and to prevent diabetes in Hispanic Americans have proved effective.^48^^,^^49^ However, such tailoring is costly, and we lack evidence of successful implementation at scale; more work can investigate the efficacy of one-size-fits-all public health efforts in lifestyle interventions across race.

We acknowledge limitations. The NHANES is a cross-sectional survey. We cannot establish temporal ordering of lifestyle behaviors and biological aging. Diet and lifestyle data were reported by participants and may be subject to social desirability bias. However, trends in responses did not change over the 2-decade period included in our study, as might be expected with an increase in public messaging about healthy lifestyle over that period. Moreover, as our focus was on differential associations of lifestyle with biological aging across race/ethnic identity groups, bias would only result if social desirability of healthy diet and physical activity differed across these groups. We are not aware of any such differences in Black and Hispanic as compared with White Americans.

Overall, this cross-sectional study of serial NHANES data from 1999-2018 found that healthier lifestyle (ie, greater adherence to a MeDi and higher level of LTPA) was associated with younger biological age across race/ethnicity. However, magnitudes of associations were larger in non-Hispanic White participants compared to non-Hispanic Black and Hispanic participants.

## Authors contributions

Talha Arif wrote the manuscript draft. Aline Thomas, Yian Gu, and Daniel W. Belsky designed and conceptualized the study and edited the manuscript. Talha Arif and Aline Thomas analyzed the data. All authors had access to all of the data, contributed to the interpretation of findings, and revised and approved the final manuscript.

## Supplementary Material

glaf194_Supplementary_Data

## Data Availability

This study analyzes publicly available data sets. Data for NHANES were obtained and are available online at: https://wwwn.cdc.gov/nchs/nhanes/.

## References

[1] Kirkwood TBL. Understanding the odd science of aging. Cell. 2005;120:437-447. 10.1016/j.cell.2005.01.027 15734677

[2] Kennedy BK , Berger SL, Brunet A, et al Geroscience: linking aging to chronic disease. Cell. 2014;159:709-713. 10.1016/j.cell.2014.10.039 25417146 PMC4852871

[3] Campisi J , Kapahi P, Lithgow GJ, Melov S, Newman JC, Verdin E. From discoveries in ageing research to therapeutics for healthy ageing. Nature. 2019;571:183-192. 10.1038/s41586-019-1365-2 31292558 PMC7205183

[4] Rolland Y , Sierra F, Ferrucci L, et al Challenges in developing Geroscience trials. Nat Commun. 2023;14:5038. 10.1038/s41467-023-39786-7 37598227 PMC10439920

[5] Belsky DW , Baccarelli AA. To promote healthy aging, focus on the environment. Nat Aging. 2023;3:1334-1344. 10.1038/s43587-023-00518-7 37946045 PMC12459207

[6] Flanagin A , Frey T, Christiansen SL; AMA Manual of Style Committee. Updated guidance on the reporting of race and ethnicity in medical and science journals. JAMA. 2021;326:621. 10.1001/jama.2021.13304 34402850

[7] Ioannidis JPA , Powe NR, Yancy C. recalibrating the use of race in medical research. JAMA. 2021;325:623. 10.1001/jama.2021.0003 33492329

[8] Raffington L , Belsky DW. Integrating DNA methylation measures of biological aging into social determinants of health research. Curr Environ Health Rep. 2022;9:196-210. 10.1007/s40572-022-00338-8 35181865

[9] Graf GH , Crowe CL, Kothari M, et al Testing Black-White disparities in biological aging among older adults in the United States: analysis of DNA-methylation and blood-chemistry methods. Am J Epidemiol. 2022;191:613-625. 10.1093/aje/kwab281 34850809 PMC9077113

[10] Cuevas AG , Cole SW, Belsky DW, McSorley AM, Shon JM, Chang VW. Multi-discrimination exposure and biological aging: results from the midlife in the United States study. Brain Behav Immun—Health. 2024;39:100774. 10.1016/j.bbih.2024.100774 39132086 PMC11315217

[11] Bonaccio M , Di Castelnuovo A, Costanzo S, et al Combined impact of healthy lifestyle factors on survival in the elderly. Eur J Public Health. 2018;28. 10.1093/eurpub/cky212.507

[12] Ng TP , Feng L, Nyunt MSZ, et al Nutritional, physical, cognitive, and combination interventions and frailty reversal among older adults: a randomized controlled trial. Am J Med. 2015;128:1225-1236.e1. 10.1016/j.amjmed.2015.06.017 26159634

[13] Waziry R , Ryan CP, Corcoran DL, et al Effect of long-term caloric restriction on DNA methylation measures of biological aging in healthy adults from the CALERIE trial [published online ahead of print February 9, 2023]. Nat Aging. 10.1038/s43587-022-00357-y PMC10148951 37118425

[14] Bischoff-Ferrari HA , Vellas B, Rizzoli R, et al Effect of vitamin D supplementation, omega-3 fatty acid supplementation, or a strength-training exercise program on clinical outcomes in older adults: the DO-HEALTH Randomized Clinical Trial. JAMA. 2020;324:1855. 10.1001/jama.2020.16909 33170239 PMC7656284

[15] De Cabo R , Mattson M. Effects of intermittent fasting on health, aging, and disease. N Engl J Med. 2020;382:298-298. 10.1056/NEJMx190038 32348665

[16] Thomas A , Belsky DW, Gu Y. Healthy lifestyle behaviors and biological aging in the U.S. National Health and Nutrition Examination Surveys 1999–2018. J Gerontol A Biol Sci Med Sci. 2023;78:1535-1542. 10.1093/gerona/glad082 36896965 PMC10460553

[17] National Health and Nutrition Examination Survey Demographic Data. https://wwwn.cdc.gov/nchs/nhanes/search/datapage.aspx?Component=Demographics

[18] Trichopoulou A , Costacou T, Bamia C, Trichopoulos D. Adherence to a Mediterranean diet and survival in a Greek population. N Engl J Med. 2003;348:2599-2608. 10.1056/NEJMoa025039 12826634

[19] Physical Activity Guidelines for Americans, 2nd ed.

[20] Ferrucci L , Gonzalez‐Freire M, Fabbri E, et al Measuring biological aging in humans: a quest. Aging Cell. 2020;19:e13080. 10.1111/acel.13080 31833194 PMC6996955

[21] Rutledge J , Oh H, Wyss-Coray T. Measuring biological age using omics data. Nat Rev Genet. 2022;23:715-727. 10.1038/s41576-022-00511-7 35715611 PMC10048602

[22] Moqri M , Herzog C, Poganik JR, et al Biomarkers of aging for the identification and evaluation of longevity interventions. Cell. 2023;186:3758-3775. 10.1016/j.cell.2023.08.003 37657418 PMC11088934

[23] Levine ME , Lu AT, Quach A, et al An epigenetic biomarker of aging for lifespan and healthspan. Aging. 2018;10:573-591. 10.18632/aging.101414 29676998 PMC5940111

[24] Kwon D , Belsky DW. A toolkit for quantification of biological age from blood chemistry and organ function test data: BioAge. GeroScience. 2021;43:2795-2808. 10.1007/s11357-021-00480-5 34725754 PMC8602613

[25] Horvath S , Raj K. DNA methylation-based biomarkers and the epigenetic clock theory of ageing. Nat Rev Genet. 2018;19:371-384. 10.1038/s41576-018-0004-3 29643443

[26] Zhang YB , Chen C, Pan XF, et al Associations of healthy lifestyle and socioeconomic status with mortality and incident cardiovascular disease: two prospective cohort studies [published online ahead of print April 14, 2021]. Br Med J. n604. 10.1136/bmj.n604 33853828 PMC8044922

[27] Hill L , Published SA. What is driving widening racial disparities in life expectancy? KFF. May 23, 2023. Accessed December 3, 2023. https://www.kff.org/racial-equity-and-health-policy/issue-brief/what-is-driving-widening-racial-disparities-in-life-expectancy/

[28] Turney IC , Lao PJ, Rentería MA, et al Brain aging among racially and ethnically diverse middle-aged and older adults. JAMA Neurol. 2023;80:73. 10.1001/jamaneurol.2022.3919 36374494 PMC9664371

[29] Diez Roux AV , Ranjit N, Jenny NS, et al Race/ethnicity and telomere length in the multi‐ethnic study of atherosclerosis. Aging Cell. 2009;8:251-257. 10.1111/j.1474-9726.2009.00470.x 19302371 PMC2713110

[30] Lao PJ , Boehme AK, Morales C, et al Amyloid, cerebrovascular disease, and neurodegeneration biomarkers are associated with cognitive trajectories in a racially and ethnically diverse, community-based sample. Neurobiol Aging. 2022;117:83-96. 10.1016/j.neurobiolaging.2022.05.004 35679806 PMC9997572

[31] Shen B , Mode NA, Noren Hooten N, et al Association of race and poverty status with DNA methylation-based age. JAMA Netw Open. 2023;6:e236340. 10.1001/jamanetworkopen.2023.6340 37027157 PMC10082406

[32] Parker D , Sloane R, Pieper CF, et al Age-related adverse inflammatory and metabolic changes begin early in adulthood. J Gerontol A Biol Sci Med Sci. 2019;74:283-289. 10.1093/gerona/gly121 29985987 PMC6376106

[33] Liu Z , Kuo PL, Horvath S, Crimmins E, Ferrucci L, Levine M. A new aging measure captures morbidity and mortality risk across diverse subpopulations from NHANES IV: a cohort study. PLOS Med. 2018;15:e1002718. 10.1371/journal.pmed.1002718 30596641 PMC6312200

[34] Sotos-Prieto M , Mattei J. Mediterranean diet and cardiometabolic diseases in racial/ethnic minority populations in the United States. Nutrients. 2018;10:352. 10.3390/nu10030352 29538339 PMC5872770

[35] Gardener H , Wright CB, Gu Y, et al Mediterranean-style diet and risk of ischemic stroke, myocardial infarction, and vascular death: the Northern Manhattan Study. Am J Clin Nutr. 2011;94:1458-1464. 10.3945/ajcn.111.012799 22071704 PMC3252546

[36] Hiza HAB , Casavale KO, Guenther PM, Davis CA. Diet quality of Americans differs by age, sex, race/ethnicity, income, and education level. J Acad Nutr Diet. 2013;113:297-306. 10.1016/j.jand.2012.08.011 23168270

[37] Gu Y , Guo J, Moshfegh AJ. Race/ethnicity and gender modify the association between diet and cognition in U.S. older adults: National Health and Nutrition Examination Survey 2011‐2014. Alzheimers Dement Transl Res Clin Interv. 2021;7:e12128. 10.1002/trc2.12128 PMC7882526 33614896

[38] Panda S , Maier G, Villareal DT. Targeting energy intake and circadian biology to engage mechanisms of aging in older adults with obesity: calorie restriction and time-restricted eating. J Gerontol A Biol Sci Med Sci. 2023;78:79-85. 10.1093/gerona/glad069 37325958 PMC10272989

[39] Arem H , Moore SC, Patel A, et al Leisure time physical activity and mortality: a detailed pooled analysis of the dose-response relationship. JAMA Intern Med. 2015;175:959. 10.1001/jamainternmed.2015.0533 25844730 PMC4451435

[40] Zhao M , Veeranki SP, Magnussen CG, Xi B. Recommended physical activity and all cause and cause specific mortality in US adults: prospective cohort study [published online July 1, 2020]Br Med J. m2031. 10.1136/bmj.m2031 32611588 PMC7328465

[41] Wen M , Li L, Su D. Physical activity and mortality among middle-aged and older adults in the United States. J Phys Act Health. 2014;11:303-312. 10.1123/jpah.2011-0281 23363569 PMC3929528

[42] Bazargan-Hejazi S , Arroyo JS, Hsia S, Brojeni NR, Pan D. A racial comparison of differences between self-reported and objectively measured physical activity among US adults with diabetes. Ethn Dis. 2017;27:403-410. 10.18865/ed.27.4.403 29225441 PMC5720950

[43] Levine ME , Crimmins EM. Evidence of accelerated aging among African Americans and its implications for mortality. Soc Sci Med. 2014;118:27-32. 10.1016/j.socscimed.2014.07.022 25086423 PMC4197001

[44] Boen CE , Yang YC, Aiello AE, et al Patterns and life course determinants of Black–White disparities in biological age acceleration: a decomposition analysis. Demography. 2023;60:1815-1841. 10.1215/00703370-11057546 37982570 PMC10842850

[45] Tajuddin SM , Hernandez DG, Chen BH, et al Novel age-associated DNA methylation changes and epigenetic age acceleration in middle-aged African Americans and Whites. Clin Epigenetics. 2019;11:119. 10.1186/s13148-019-0722-1 31426852 PMC6700815

[46] Crimmins EM , Thyagarajan B, Levine ME, Weir DR, Faul J. Associations of age, sex, race/ethnicity, and education with 13 epigenetic clocks in a nationally representative U.S. sample: the Health and Retirement Study. J Gerontol A Biol Sci Med Sci. 2021;76:1117-1123. 10.1093/gerona/glab016 33453106 PMC8140049

[47] Levine ME , Crimmins EM. A genetic network associated with stress resistance, longevity, and cancer in humans. J Gerontol A Biol Sci Med Sci. 2016;71:703-712. 10.1093/gerona/glv141 26355015 PMC4888382

[48] Brewer LC , Balls-Berry JE, Dean P, Lackore K, Jenkins S, Hayes SN. Fostering African-American Improvement in Total Health (FAITH!): an application of the American Heart Association’s Life’s Simple 7^TM^ among Midwestern African-Americans. J Racial Ethn Health Disparities. 2017;4:269-281. 10.1007/s40615-016-0226-z 27059054 PMC5516637

[49] Ockene IS , Tellez TL, Rosal MC, et al Outcomes of a Latino community-based intervention for the prevention of diabetes: the Lawrence Latino Diabetes Prevention Project. Am J Public Health. 2012;102:336-342. 10.2105/AJPH.2011.300357 22390448 PMC3483988

