# Supplementary material for: Racial/ethnic differences in the association of lifestyle factors with biological aging in NHANES, 1999-2018
Source: J Gerontol A Biol Sci Med Sci. 2025 Sep 10;80(10):glaf194. doi: 10.1093/gerona/glaf194 (PMC12483585; doi:10.1093/gerona/glaf194)
Supplement: glaf194_Supplementary_Data [file glaf194_supplementary_data.docx]

**Supplementary Material**

**eFigure 1 – Page 1**

**eFigure 2 – Page 2**

**eFigure 3 – Page 3**

**eFigure 4 – Page 4**

**eTable 1 – Page 5**

**eTable 2 – Page 6-7**

**eTable 3 – Page 8-9**

**eFigure 5 – Page 10**

**eFigure 6 – Page 11**

**eTable 4 – Page 12**


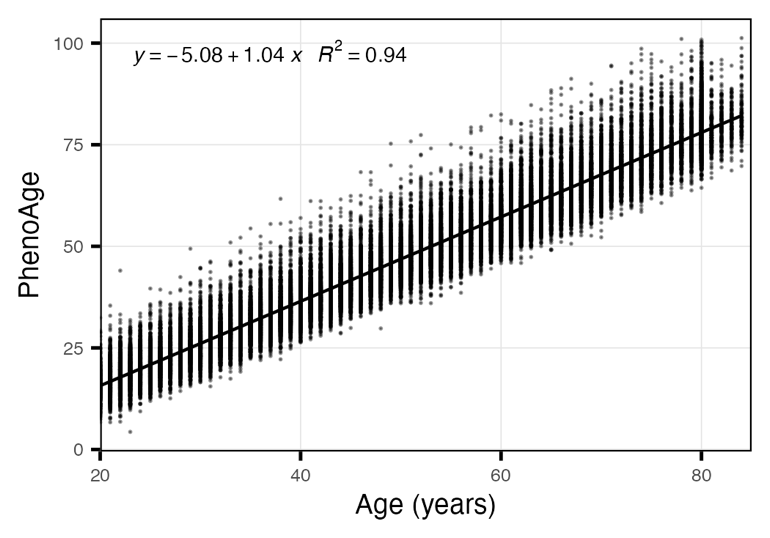


**eFigure 1. Correlation of PhenoAge and chronological age of study population (aged ≥20 and <85 years), NHANES 1999-2018 (n = 42,625).**


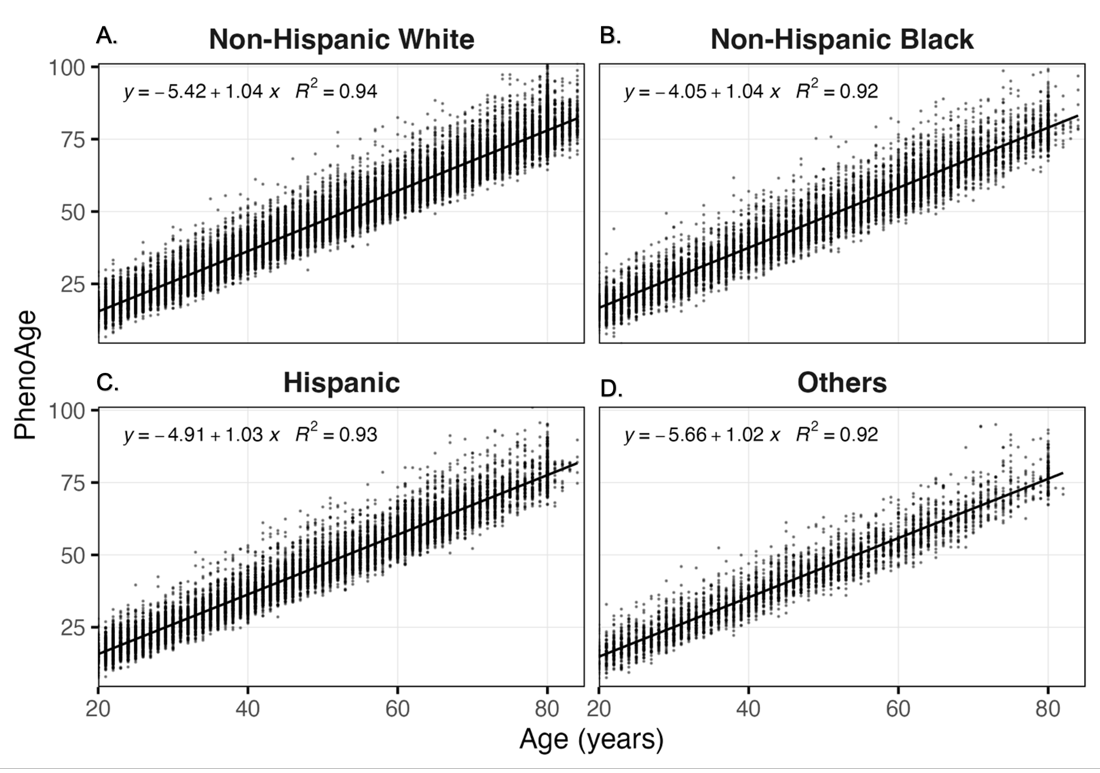


**eFigure 2. Correlation of PhenoAge and chronological age of study population by race, NHANES 1999-2018 (n = 42,625).**

**
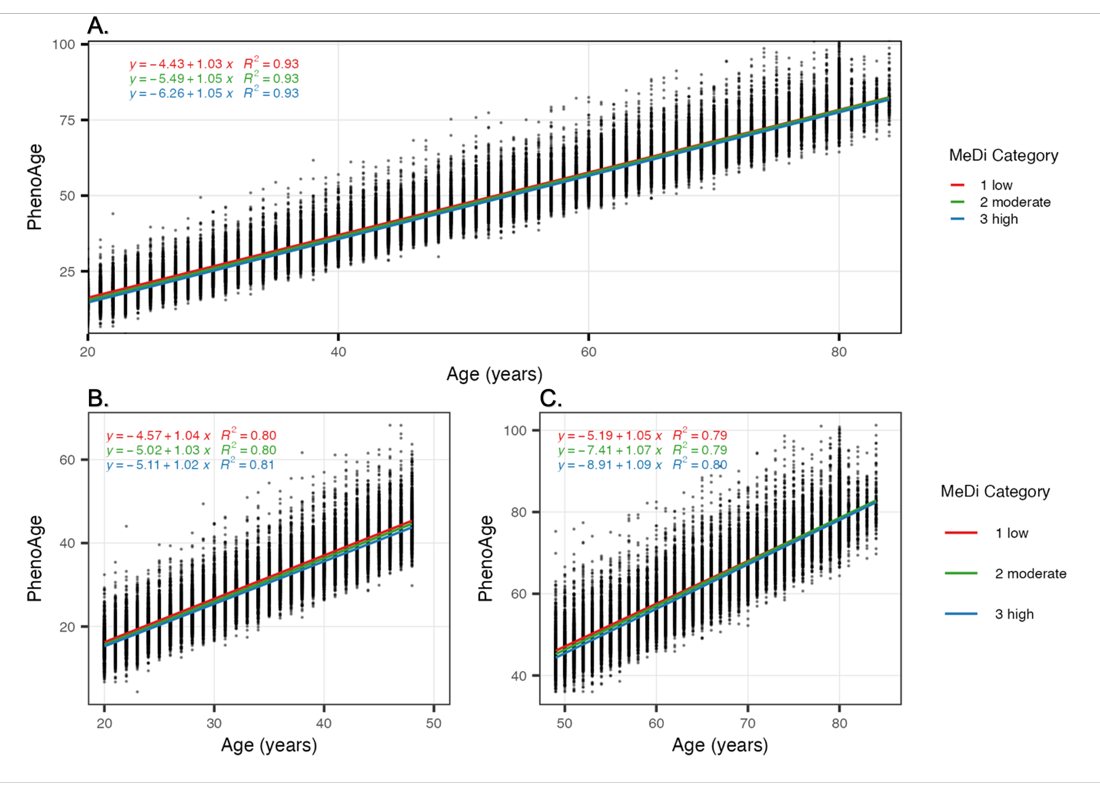
**

**eFigure 3. Correlation of PhenoAge and chronological age of study population by MeDi category, NHANES 1999-2018 (n = 42,625).**

Panel A demonstrates the correlation across the entire population. Panel B (bottom left) includes younger participants (age 20 to 48). Panel C (bottom right) includes older participants (age 49 to 85).

**
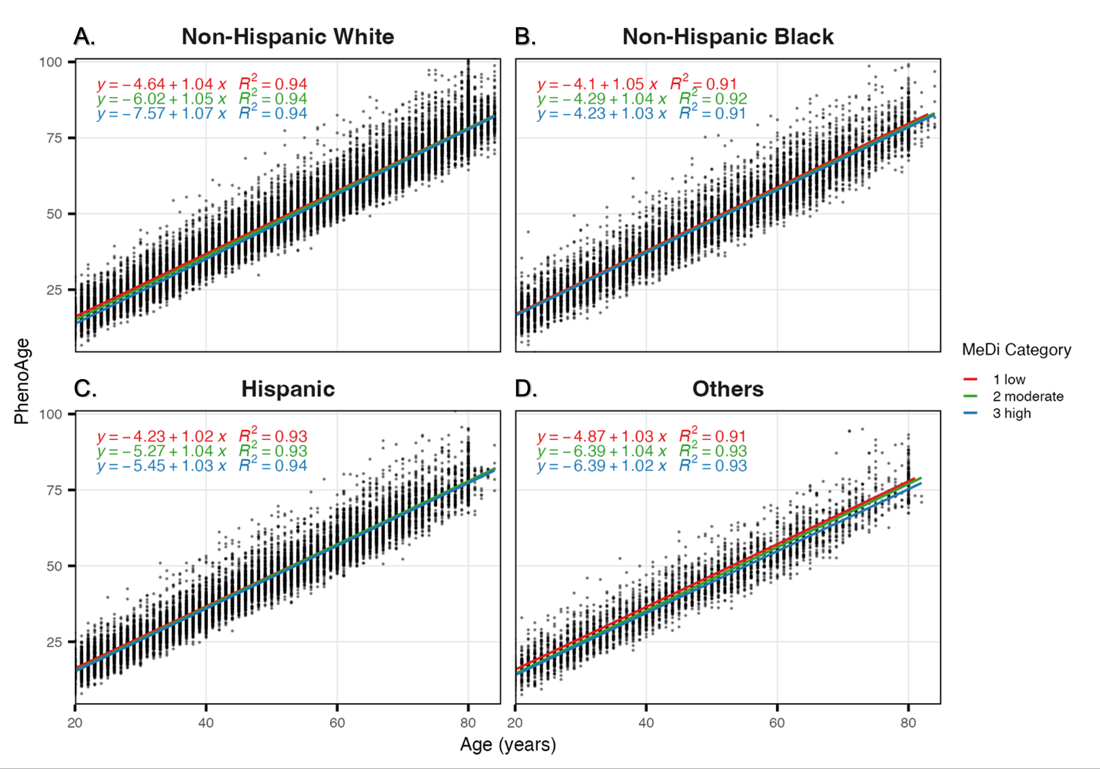
**

**eFigure 4. Correlation of PhenoAge and chronological age of study population by MeDi category and race, NHANES 1999-2018 (n = 42,625).**

**eTable 1. Associations of MeDi with PhenoAge advancement, by race/ethnicity group, estimated by adjusted linear regressions, NHANES 1999-2018**

|  | | **Non-Hispanic White** | | **Non-Hispanic Black** | **Hispanic** | **Other** |
| --- | --- | --- | --- | --- | --- | --- |
| 1. **β (95% CI) for MeDi score on PhenoAge** | | | | | | |
| Model 1 (demographics) | | -0.07 (-0.08; -0.06)**^*^** | | -0.04 (-0.05; -0.02)**^*^** | -0.05 (-0.06; -0.04)**^*^** | -0.12 (-0.15; -0.09)**^*^** |
| Model 2 (socioeconomic status) | -0.04 (-0.05; -0.03)^*^ | | -0.02 (-0.03; -0.001)^*^ | | -0.03 (-0.05; -0.02)**^*^** | -0.07 (-0.09; -0.04)**^*^** |
| Model 3 (comorbidities) | | -0.06 (-0.07; -0.05)**^*^** | | -0.05 (-0.06; -0.03)**^*^** | -0.05 (-0.06; -0.03)**^*^** | -0.11 (-0.14; -0.08)**^*^** |
| Model 4 (LTPA) | | -0.06 (-0.07; -0.05)**^*^** | | -0.03 (-0.05; -0.02)**^*^** | -0.05 (-0.06; -0.04)**^*^** | -0.12 (-0.15; -0.09)**^*^** |
| 1. **β (95% CI) for MeDi-by-race/ethnicity interaction on PhenoAge** | | | | | | |
| Model 1 (demographics) | | Ref. | | 0.03 (0.01; 0.05)^*^ | 0.02 (0.003; 0.04)^*^ | -0.05 (-0.08; -0.02)^*^ |
| Model 2 (socioeconomic status) | | Ref. | | 0.02 (-0.0001; 0.04)^**^ | 0.002 (-0.02; 0.02) | -0.03 (-0.06; -0.001)^*^ |
| Model 3 (comorbidities) | | Ref. | | 0.02 (-0.003; 0.04) | 0.02 (-0.001; 0.04) | -0.05 (-0.08; -0.02)^*^ |
| Model 4 (LTPA) | | Ref. | | 0.03 (0.01; 0.05)^*^ | 0.01 (-0.01; 0.03) | -0.06 (-0.09; -0.03)^*^ |

Abbreviations: CI = confidence interval, LTPA = leisure-time physical activity, MeDi = Mediterranean diet

**^*^** Represents p value < 0.05.

**^**^** Represents p value = 0.054

Panel A presents β coefficients and 95% CIs for the associations of MeDi score with PhenoAge advancement, in all race/ethnicity group.

Panel B presents β coefficients and 95% CIs for the interaction testing the product of MeDi score by race/ethnicity (with Non-Hispanic White as reference) to evaluated whether the association of MeDi with PhenoAge advancement differed across race/ethnicity groups.

PhenoAge advancement was standardized so that regression coefficients are relative to 1 SD (= 4.6 years) of PhenoAge advancement. MeDi score was taken as a continuous variable (for 1-point increase). Models were run on participants without missing data for covariates. Both models were adjusted for age, sex, total energy intake, and NHANES wave. Model 2 was additionally adjusted for socioeconomic and lifestyle factors (education, income-to-poverty ratio, marital status, smoking, and body mass index) (n = 38,771). Model 3 was additionally adjusted for history of 10 comorbidities (i.e. histories of diabetes, hypertension, hypercholesterolemia, stroke, cardiovascular disease, chronic bronchitis, liver condition, pulmonary emphysema, thyroid disease, and arthritis) (n = 33,519). Model 4 was additionally adjusted for LTPA (n = 42,625).

**eTable 2. Associations of LTPA level with PhenoAge advancement, by race/ethnicity group, estimated by adjusted linear regressions, NHANES 1999-2018**

|  | **Non-Hispanic White** | | | | | **Non-Hispanic Black** | | | **Hispanic** | | **Other** | |
| --- | --- | --- | --- | --- | --- | --- | --- | --- | --- | --- | --- | --- |
| 1. **β (95% CI) for LTPA level on PhenoAge^*^** | | | | | | | | | | | | |
| Model 1 (demographics) | | | | |  | |  | | |  | |  |
| Sedentary | | Ref. | | | | Ref. | | | Ref. | | | Ref. |
| Low | | | | -0.25 (-0.29; -0.21)^*^ | | | | -0.11 (-0.18; 0.04)^*^ | | -0.13 (-0.19; -0.06)^*^ | | -0.15 (-0.29; -0.02)^*^ |
| Moderate | | | | -0.34 (-0.39; -0.28)^*^ | | | | -0.19 (-0.29; -0.10)^*^ | | -0.20 (-0.27; -0.12)^*^ | | -0.21 (-0.36; -0.05)^*^ |
| High | | | | -0.37 (-0.41; -0.33)^*^ | | | | -0.22 (-0.28; -0.16)^*^ | | -0.14 (-0.20, -0.09)^*^ | | -0.20 (-0.30; -0.10)^*^ |
| Model 2 (socioeconomic status) | | | | | | | | | | | | |
| Sedentary | Ref. | | | | | Ref. | | | Ref. | | Ref. | |
| Low | -0.14 (-0.18; -0.10)^*^ | | | | | -0.04 (-0.11; 0.04) | | | -0.07 (-0.14; 0.002) | | -0.07 (-0.20; 0.07) | |
| Moderate | -0.17 (-0.22; -0.11)^*^ | | | | | -0.13 (-0.22; -0.03)^*^ | | | -0.07 (-0.13; -0.01)^*^ | | -0.17 (-0.28; -0.05)^*^ | |
| High | -0.16 (-0.20; -0.12)^*^ | | | | | -0.08 (-0.14; -0.02)^*^ | | | -0.02 (-0.08; 0.03) | | -0.09 (-0.18; 0.01) | |
| Model 3 (comorbidities) | | | | | | | | | | | | |
| Sedentary | Ref. | | | | | Ref. | | | Ref. | | Ref. | |
| Low | -0.21 (-0.25; -0.17)^*^ | | | | | -0.11 (-0.20; -0.02)^*^ | | | -0.13 (-0.20; -0.05)^*^ | | -0.12 (-0.25; 0.02)^*^ | |
| Moderate | -0.25 (-0.30; -0.20)^*^ | | | | | -0.20 (-0.28; -0.11)^*^ | | | -0.18 (-0.26; -0.10)^*^ | | -0.17 (-0.30; -0.04)^*^ | |
| High | -0.30 (-0.34; -0.26)^*^ | | | | | -0.19 (-0.25; -0.12)^*^ | | | -0.12 (-0.19; -0.06)^*^ | | -0.09 (-0.19; 0.02)^*^ | |
| Model 4 (MeDi score) | | | | | | | | | | | | |
| Sedentary | Ref. | | | | | Ref. | | | Ref. | | Ref. | |
| Low | -0.24 (-0.28; -0.19)^*^ | | | | | -0.10 (-0.17; -0.04)^*^ | | | -0.13 (-0.20; -0.06)^*^ | | -0.14 (-0.27; -0.01)^*^ | |
| Moderate | -0.32 (-0.37; -0.27)^*^ | | | | | -0.18 (-0.27; -0.09)^*^ | | | -0.19 (-0.27; -0.12)^*^ | | -0.19 (-0.34; -0.05)^*^ | |
| High | -0.34 (-0.38; -0.30)^*^ | | | | | -0.20 (-0.26; -0.15)^*^ | | | -0.14 (-0.19; -0.08)^*^ | | -0.19 (-0.29; -0.10)^*^ | |
| 1. **β (95% CI) for LTPA-by-race/ethnicity interaction on PhenoAge** | | | | | | | | | | | | |
| Model 1 (demographics) | | | | | | | | | | | | |
| Sedentary | Ref. | | | | | Ref. | | | Ref. | | Ref. | |
| Low | Ref. | | | | | 0.14 (0.06; 0.23)^*^ | | | 0.13 (0.05; 0.21)^*^ | | 0.10 (-0.04; 0.24) | |
|  | **Non-Hispanic White** | | | | | **Non-Hispanic Black** | | | **Hispanic** | | **Other** | |
| 1. **β (95% CI) for LTPA level on PhenoAge^*^** | | | | | | | | | | | | |
| Moderate | Ref. | | | | | 0.14 (0.04; 0.24)^*^ | | | 0.14 (0.05; 0.23)^*^ | | 0.13 (-0.03; 0.29) | |
| High | Ref. | | | | | 0.15 (0.08; 0.22)^*^ | | | 0.23 (0.15; 0.30)^*^ | | 0.17 (0.06; 0.27)^*^ | |
| Model 2 (socioeconomic status) | | | | | | | | | | | | |
| Sedentary | Ref. | | | | | Ref. | | | Ref. | | Ref. | |
| Low | Ref. | | | | | 0.10 (0.01; 0.20)^*^ | | | 0.07 (-0.01; 0.15) | | 0.07 (-0.07; 0.22) | |
| Moderate | Ref. | | | | | 0.04 (-0.06; 0.14) | | | 0.10 (0.01; 0.18)^*^ | | 0.003 (-0.13; 0.13) | |
| High | Ref. | | | | | 0.08 (0.01; 0.15)^*^ | | | 0.14 (0.06; 0.21)^*^ | | 0.07 (-0.03; 0.17) | |
| Model 3 (comorbidities) | | |  | | | |  | | |  | |  |
| Sedentary | Ref. | | | | | Ref. | | | Ref. | | | Ref. |
| Low | Ref. | | | | | 0.10 (-0.01; 0.20) | | | 0.08 (-0.001; 0.17) | | | 0.09 (-0.05; 0.23) |
| Moderate | Ref. | | | | | 0.05 (-0.04; 0.14) | | | 0.07 (-0.03; 0.17) | | | 0.08 (-0.05; 0.22) |
| High | Ref. | | | | | 0.11 (0.04; 0.19)^*^ | | | 0.18 (0.10; 0.25)^*^ | | | 0.21 (0.11; 0.32)^*^ |
| Model 4 (MeDi score) | | | | | | | | | | | | |
| Sedentary | Ref. | | | | | Ref. | | | Ref. | | Ref. | |
| Low | Ref. | | | | | 0.13 (0.05; 0.22)^*^ | | | 0.11 (0.03; 0.19)^*^ | | 0.09 (-0.04; 0.23) | |
| Moderate | Ref. | | | | | 0.14 (0.04; 0.23)^*^ | | | 0.12 (0.03; 0.21)^*^ | | 0.12 (-0.03; 0.28) | |
| High | Ref. | | | | | 0.14 (0.07; 0.20)^*^ | | | 0.20 (0.13; 0.27)^*^ | | 0.14 (0.04; 0.25)^*^ | |

Abbreviations: CI = confidence interval, LTPA = leisure-time physical activity, MET = metabolic equivalent of task

**^*^** Represents p value < 0.05.

Panel A presents β coefficients and 95% CIs for the associations of LTPA with PhenoAge advancement, in all race/ethnicity groups.

Panel B presents β coefficients and 95% CIs for the interaction testing the product of LTPA levels by race/ethnicity (with Non-Hispanic White as reference) to evaluated whether the association of LTPA with PhenoAge advancement differed across race/ethnicity groups.

PhenoAge advancement was standardized so that regression coefficients are relative to 1 SD (= 4.6 years) of PhenoAge advancement. LTPA levels were defined as: sedentary (0 MET min/week; reference), low (1 to 500 MET min/week), moderate (500 to 1000 MET min/week), and high (>1000 MET min/week). Models were run on participants without missing data for covariates. Both models were adjusted for age, sex, total energy intake, and NHANES wave. Model 2 was additionally adjusted for socioeconomic and lifestyle factors (education, income-to-poverty ratio, marital status, smoking, and body mass index) (n = 38,771). Model 3 was additionally adjusted for history of 10 comorbidities (i.e. histories of diabetes, hypertension, hypercholesterolemia, stroke, cardiovascular disease, chronic bronchitis, liver condition, pulmonary emphysema, thyroid disease, and arthritis) (n = 33,519). Model 4 was additionally adjusted for MeDi score (n = 42,625).

**eTable 3. Associations of MeDi and LTPA with PhenoAge advancement, by race/ethnicity group, estimated by adjusted linear regressions, among participants without history of chronic disease, NHANES 1999-2018 (n = 10,682)**

|  | **Non-Hispanic White** | | **Non-Hispanic Black** | | **Hispanic** | | **Other** | |
| --- | --- | --- | --- | --- | --- | --- | --- | --- |
| 1. **β (95% CI) and p values for MeDi score and LTPA level on PhenoAge** | | | | | | | | |
|  | β (95% CI) | p | β (95% CI) | p | β (95% CI) | p | β (95% CI) | p |
| MeDi score |  |  |  |  |  |  |  |  |
| For 1-point | -0.06 (-0.08; -0.05) | <0.001 | -0.04 (-0.07; -0.01) | 0.01 | -0.02 (-0.04; 0.001) | 0.06 | -0.09 (-0.12; -0.05) | <0.001 |
| LTPA level |  |  |  |  |  |  |  |  |
| Sedentary | Ref. |  | Ref. |  | Ref. |  | Ref. |  |
| Low | -0.23 (-0.31; -0.15) | <0.001 | 0.03 (-0.15; 0.21) | 0.74 | -0.12 (-0.25; 0.01) | 0.07 | -0.11 (-0.26; 0.04) | 0.16 |
| Moderate | -0.29 (-0.38; -0.21) | <0.001 | -0.15 (-0.31; 0.02) | 0.08 | -0.11 (-0.24; 0.01) | 0.08 | -0.07 (-0.22; 0.09) | 0.38 |
| High | -0.28 (-0.35; -0.22) | <0.001 | -0.12 (-0.23; -0.01) | 0.03 | -0.11 (-0.19, -0.03) | 0.005 | -0.04 (-0.15; 0.08) | 0.52 |
| 1. **β (95% CI) and P values for MeDi-by-race/ethnicity and LTPA-by- race/ethnicity interactions on PhenoAge** | | | | | | | | |
|  |  |  | β (95% CI) | p | β (95% CI) | p | β (95% CI) | p |
| MeDi score |  |  |  |  |  |  |  |  |
| For 1-point | Ref. |  | 0.03 (-0.01; 0.06) | 0.12 | 0.04 (0.01; 0.07) | 0.005 | -0.02 (-0.06; 0.01) | 0.21 |
| LTPA level |  |  |  |  |  |  |  |  |
| Sedentary | Ref. |  | Ref. |  | Ref. |  | Ref. |  |
| Low | Ref. |  | 0.26 (0.07; 0.46) | 0.01 | 0.11 (-0.03; 0.25) | 0.12 | 0.12 (-0.04; 0.29) | 0.14 |
| Moderate | Ref. |  | 0.15 (-0.02; 0.31) | 0.08 | 0.18 (0.04; 0.33) | 0.02 | 0.23 (0.05; 0.40) | 0.01 |
| High | Ref. |  | 0.16 (0.03; 0.29) | 0.02 | 0.17 (0.06; 0.27) | 0.002 | 0.24 (0.11; 0.37) | <0.001 |

Abbreviations: LTPA = leisure-time physical activity, MeDi = Mediterranean diet, MET = metabolic equivalent of task

Panel A presents β coefficients and 95% confidence intervals for the associations between MeDi score or LTPA level and PhenoAge advancement, in all race/ethnicity group.

Panel B presents β coefficients and 95% CIs for the interaction testing the product of MeDi or LTPA by race/ethnicity (with Non-Hispanic White [NHW] as reference) to evaluate whether the associations of MeDi and LTPA with PhenoAge advancement differed across race/ethnicity groups. ꞵ coefficients for the interaction of race/ethnicity with MeDi indicates the difference of the MeDi-PhenoAge association between each of the other race/ethnicity groups and the NHW (reference) group. ꞵ coefficients for the interaction of race/ethnicity with LTPA indicate the difference of the LTPA-PhenoAge association between each of the other race/ethnicity groups and the NHW (reference) group.

PhenoAge advancement was standardized so that regression coefficients are relative to 1 SD (= 4.6 years) of PhenoAge advancement. MeDi score was taken as a continuous variable and LTPA levels were defined as: sedentary (0 MET min/week; reference), low (1 to 500 MET min/week), moderate (500 to 1000 MET min/week), and high (>1000 MET min/week). Models adjusted for age, sex, total energy intake, and NHANES wave.

**
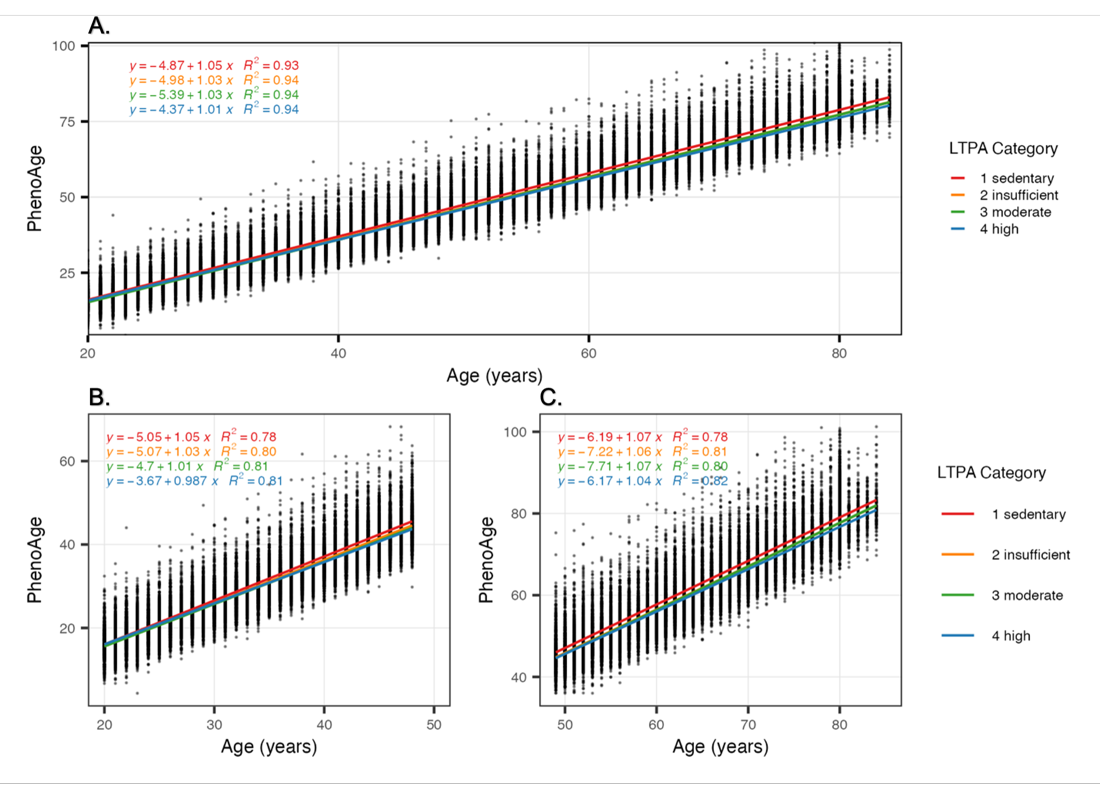
**

**eFigure 5. Correlation of PhenoAge and chronological age of study population by LTPA category, NHANES 1999-2018 (n = 42,625).**

Panel A demonstrates the correlation across the entire population. Panel B (bottom left) includes younger participants (age 20 to 49). Panel C (bottom right) includes older participants (age 49 to 85).

**
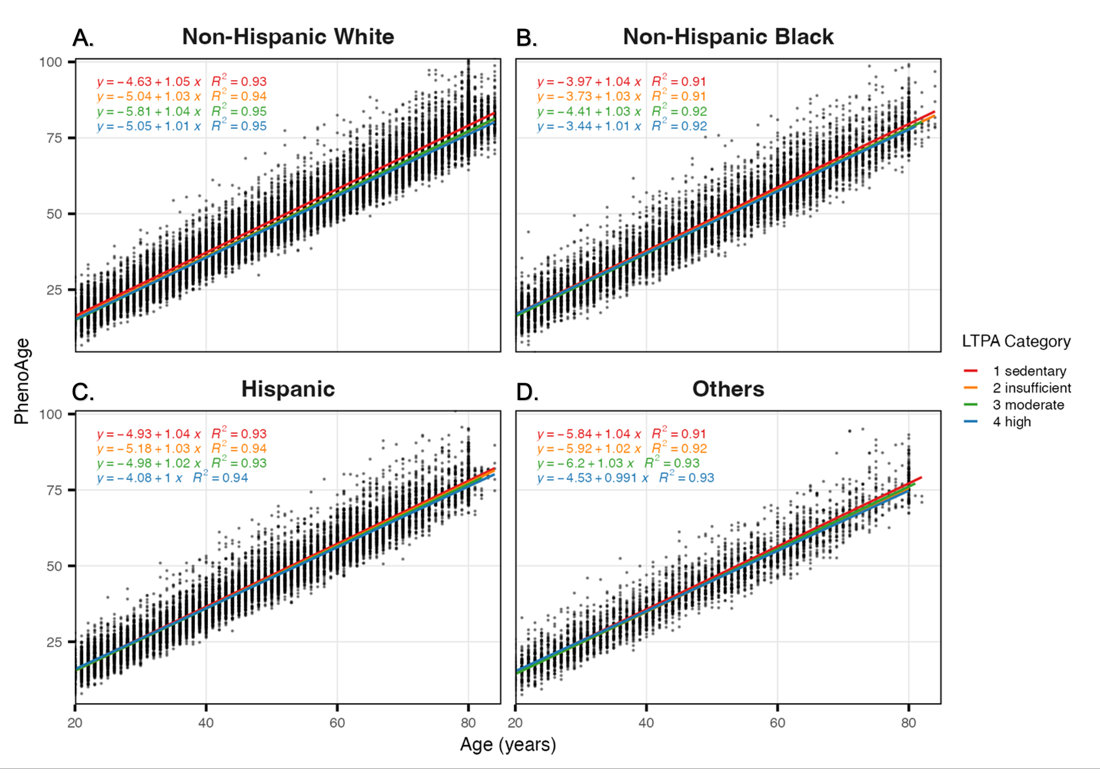
**

**eFigure 6. Correlation of PhenoAge and chronological age of study population by LTPA category and race, NHANES 1999-2018 (n = 42,625).**

**eTable 4. Associations of binary MeDi and binary LTPA with PhenoAge advancement, by race/ethnicity group, estimated by adjusted linear regressions, NHANES 1999-2018 (n = 42,625)**

|  | **Non-Hispanic White** |  | **Non-Hispanic Black** |  | **Hispanic** |  | **Other** |  |
| --- | --- | --- | --- | --- | --- | --- | --- | --- |
|  | β (95% CI) | p | β (95% CI) | p | β (95% CI) | p | β (95% CI) | p |
| MeDi adherence (moderate/high vs low) | -0.18 (-0.21; -0.14) | <0.001 | -0.10 (-0.16; -0.04) | 0.001 | -0.11 (-0.16; -0.07) | <0.001 | -0.36 (-0.47; -0.26) | <0.001 |
| LTPA level  (some vs sedentary) | -0.33 (-0.36; -0.29) | <0.001 | -0.18 (-0.23; -0.13) | <0.001 | -0.15 (-0.19; -0.10) | <0.001 | -0.19 (-0.28; -0.09) | <0.001 |
| MeDi adherence (moderate/high vs low) | Ref. |  | 0.08 (0.01; 0.14) | 0.03 | 0.06 (-0.003; 0.12) | 0.06 | -0.19 (-0.30; -0.08) | <0.001 |
| LTPA level  (some vs sedentary) | Ref. |  | 0.14 (0.09; 0.20) | <0.001 | 0.18 (0.12; 0.23) | <0.001 | 0.14 (0.04; 0.24) | 0.01 |

Abbreviations: LTPA = leisure-time physical activity, MeDi = Mediterranean diet, MET = metabolic equivalent of task

Panel A presents β coefficients and 95% confidence intervals for the associations between adherence to MeDi or LTPA level and PhenoAge advancement, in all race/ethnicity group.

Panel B presents β coefficients and 95% CIs for the interaction testing the product of MeDi or LTPA by race/ethnicity (with Non-Hispanic White as reference) to evaluated whether the associations of MeDi and LTPA with PhenoAge advancement differed across race/ethnicity groups. ꞵ coefficients for the interaction of race/ethnicity with MeDi indicates the difference of the MeDi-PhenoAge association between each of the other race/ethnicity groups and the NHW (reference) group. ꞵ coefficients for the interaction of race/ethnicity with LTPA indicate the difference of the LTPA-PhenoAge association between each of the other race/ethnicity groups and the NHW (reference) group.

PhenoAge advancement was standardized so that regression coefficients are relative to 1 SD (= 4.6 years) of PhenoAge advancement. Adherence to MeDi was dichotomized by combining the moderate and high adherence categories (score 4 to 9) vs low adherence (score 0 to 3, reference). LTPA levels were categorized as ‘some LTPA’ (>0 MET min/week) vs sedentary (0 MET min/week; reference). Models adjusted for age, sex, total energy intake, and NHANES wave.
